# Rare Association of Non-Bacterial Thrombotic Endocarditis, Myocardial Infarction, and Acute Limb Ischemia Secondary to Rheumatoid Arthritis: Comprehensive Case Series With Literature Review

**DOI:** 10.7759/cureus.13319

**Published:** 2021-02-12

**Authors:** Raul Angel Garcia, Nirmal Guragai, Rahul Vasudev, Preet Randhawa, Mirette G Habib

**Affiliations:** 1 Cardiology, Trinitas Regional Medical Center, Elizabeth, USA; 2 Cardiology, Saint Joseph's Regional Medical Center, Paterson, USA; 3 Cardiology, Saint Joseph's University Medical Center, Paterson, USA; 4 Cardiology, Saint Michael's Medical Center, Newark, USA

**Keywords:** non-bacterial thrombotic endocarditis, non-st elevation myocardial infraction, rheumatoid arthriitis, limb ischemia

## Abstract

Most cases of non-bacterial thrombotic endocarditis (NBTE) tend to be related to malignancy or rheumatologic and autoimmune disorders like systemic lupus erythematosus. Rheumatoid arthritis (RA) itself has been associated with increased atherosclerosis, coronary artery plaque formation, and endothelial damage. However, it is rare to see NBTE in RA, simultaneously presenting with the acute coronary syndrome and acute limb ischemia due to distant embolization. Here we present a case of a 46-year-old female presenting with chest pain and right leg numbness, found to have ST-elevation myocardial infarction (STEMI) and occlusion of a peripheral artery due to embolization of vegetation present in the aortic valve. We also provide an extensive literature review of the relationship between NBTE and MI. One must be extra vigilant in managing these patients, especially if the size of vegetation is large as it has a tendency to embolize causing devastating complications.

## Introduction

Most cases of non-bacterial thrombotic endocarditis (NBTE) tend to be related to malignancy. Other unusual causes include rheumatologic and autoimmune disorders like systemic lupus erythematosus (SLE), antiphospholipid syndrome, rheumatic heart disease, HIV, and more. Rheumatoid arthritis (RA) has been associated with NBTE as well as increased atherosclerosis, coronary artery plaque formation, and endothelial damage [1]. These lead to increase risk in coronary artery disease including myocardial infarction, stroke, and thromboembolic disease. However, it is rare for patients with RA to simultaneously present with NBTE, ST-elevation myocardial infarction (STEMI), and acute limb ischemia. Here we present a case of STEMI secondary to thromboembolic phenomena from NBTE. We review the cardiovascular complications of RA and provide an extensive literature review of the relationship between NBTE and MI.

## Case presentation

**Figure 1:**
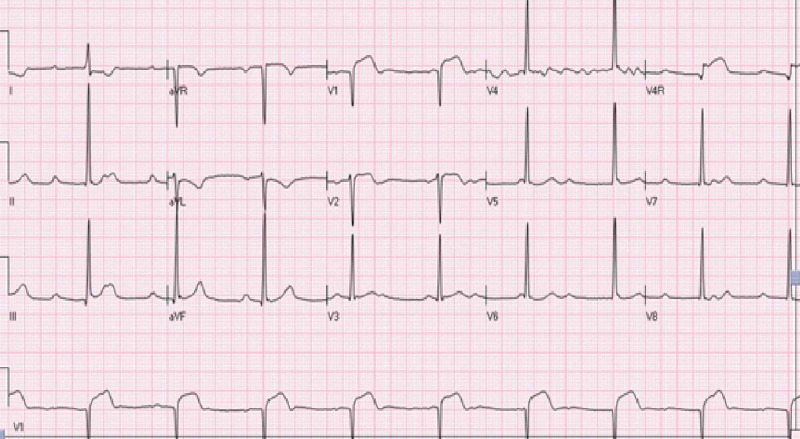
Initial EKG (while symptomatic) showing complete heart block with junctional escape rhythm and ST elevation in anterior precordial leads with ST depression in leads I and aVL EKG: electrocardiogram

A 46 years old female with a history of unspecified arthritis presented to the emergency department with complaints of substernal chest pain and right leg numbness of one-hour duration. The chest pain was radiating to the left shoulder and was associated with diaphoresis and dyspnea. On examination, vitals revealed a blood pressure of 92/69 mmHg, heart rate of 54/min, oxygen saturation of 100% on room air, and afebrile at 98.3°F. There was a soft systolic murmur in the right upper sternal border. Pulsation on dorsalis pedis and posterior tibial artery and sensation on right lower extremity was decreased. Electrocardiogram (EKG) revealed complete heart block with junctional escape rhythm and ST elevation in anterior precordial leads with ST depression in leads I and aVL (Figure 1). Pertinent labs revealed initial troponin-I of 0.05 ng/ml. The remaining blood work, including complete blood count and the basic metabolic panel, was unremarkable.

**Figure 2:**
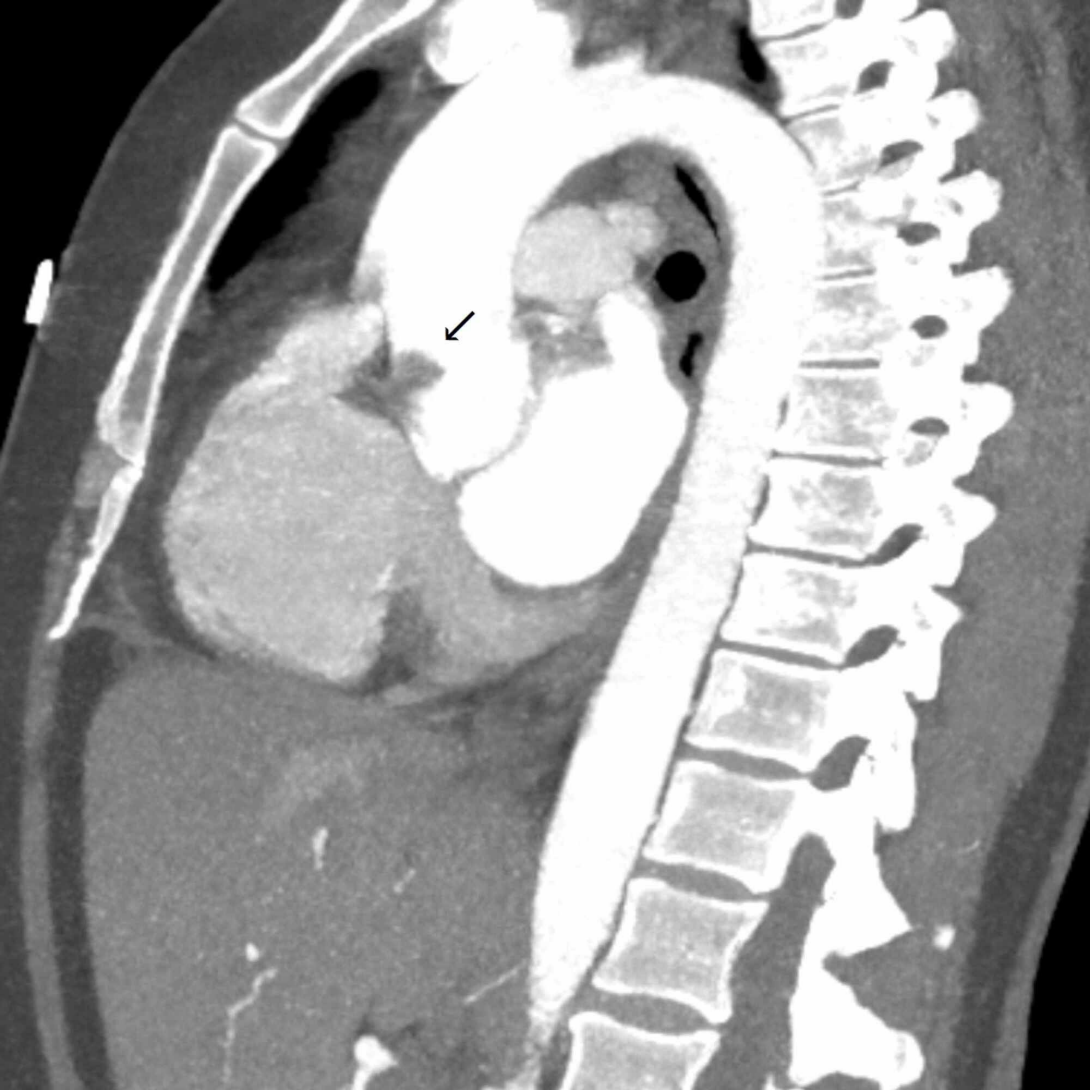
Computerized tomography of chest with angiography showing filling defect (arrow) at the root of the aorta

**Figure 3:**
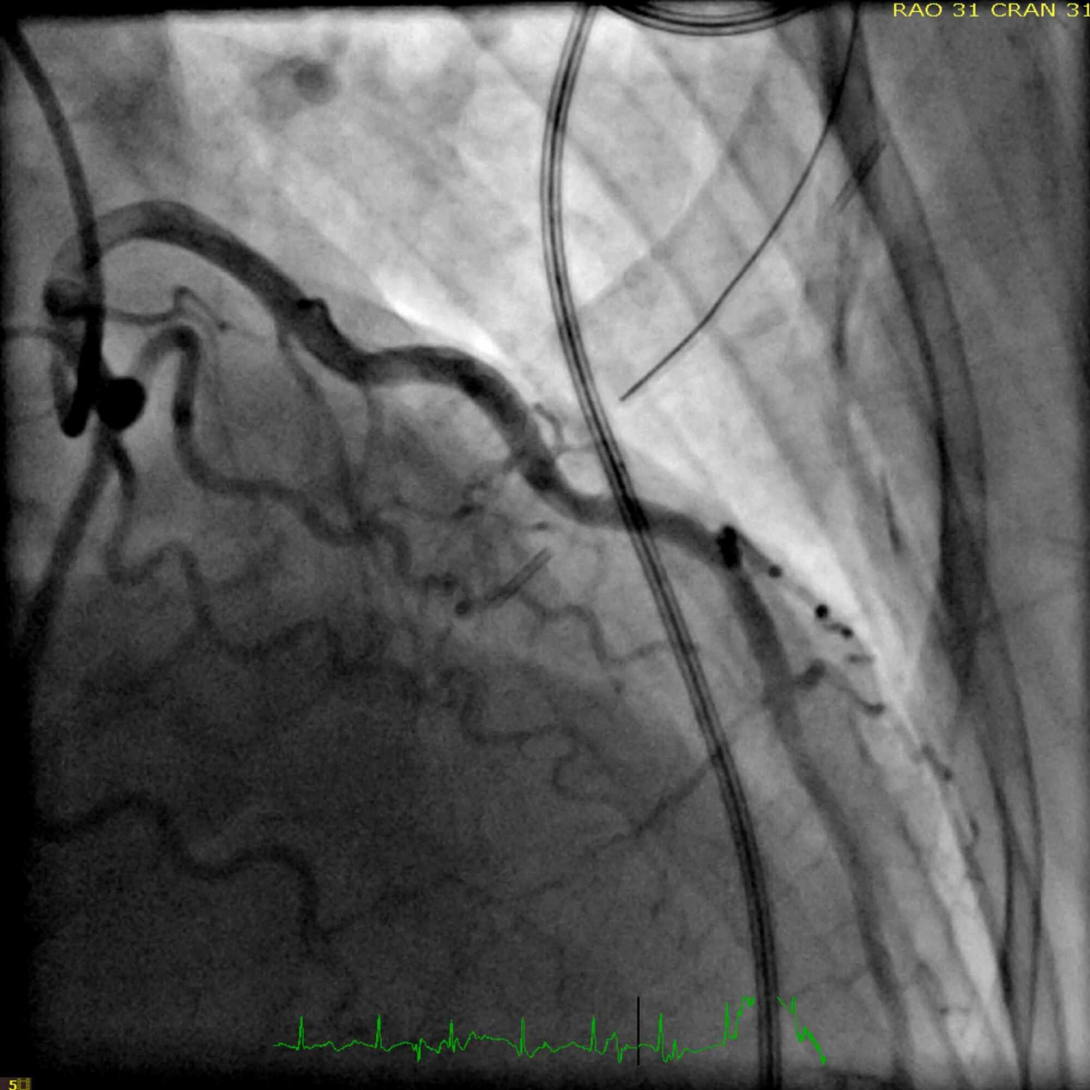
Left heart cardiac catheterization showing non-obstructive left coronary artery

**Figure 4:**
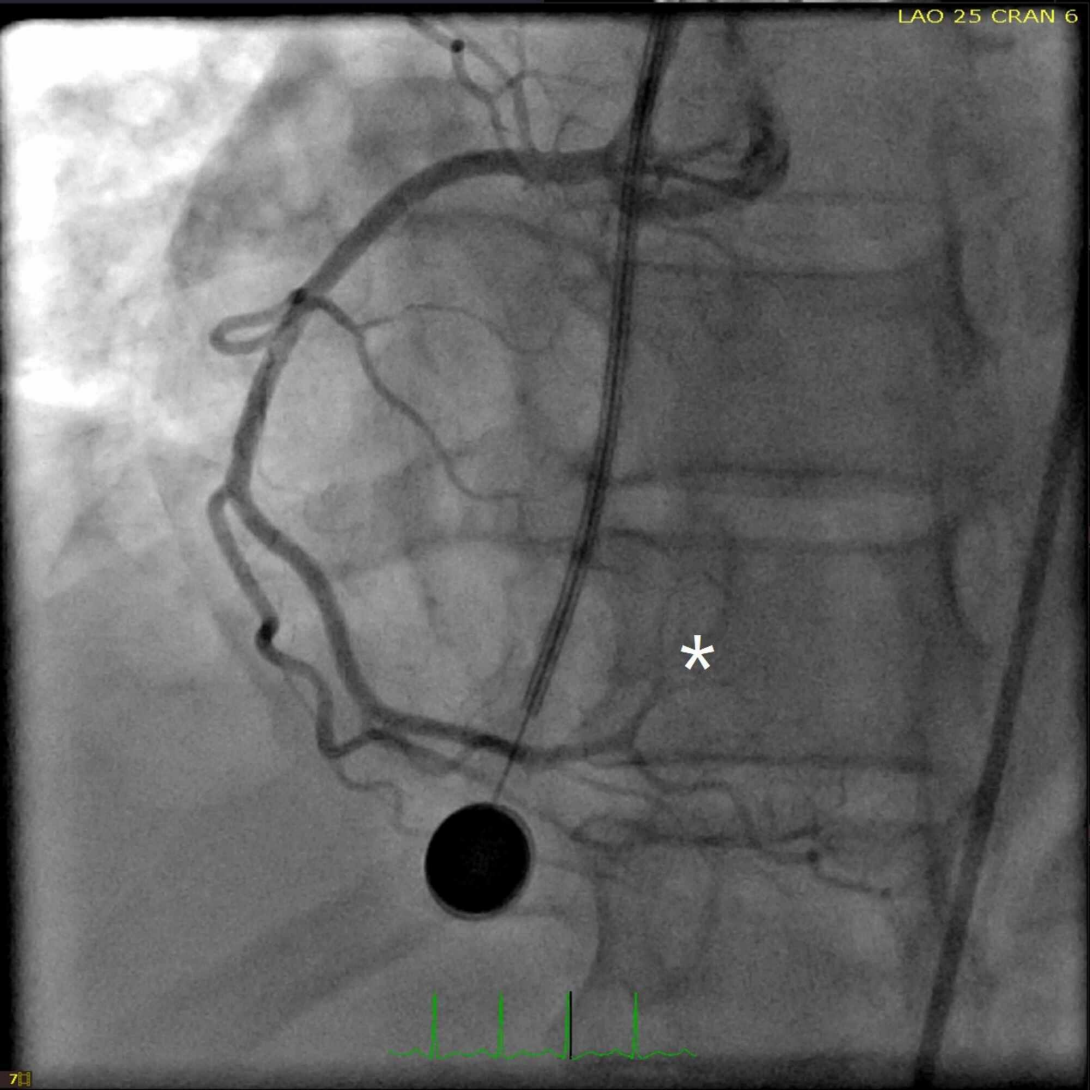
Left heart cardiac catheterization revealing non-obstructive right coronary artery except complete occlusion with filling defect probably secondary to distal thrombus noted in the posterior left ventricular branch of right coronary artery (white asterisk)

**Figure 5:**
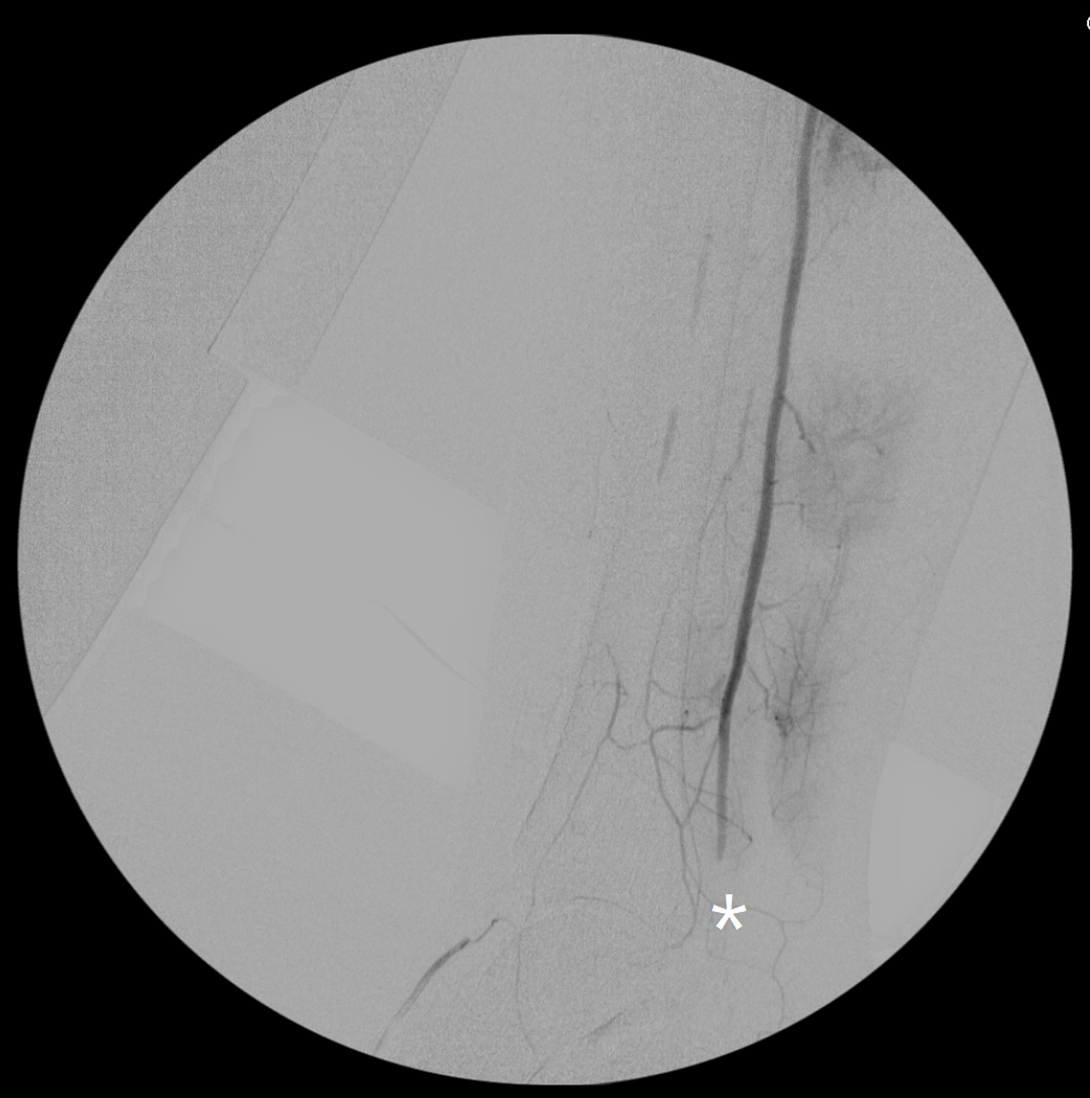
Peripheral angiogram showing complete occlusion of distal right anterior tibial artery (asterisk) and absence of right posterior tibial artery (due to complete occlusion proximally, not shown in the image)

**Figure 6:**
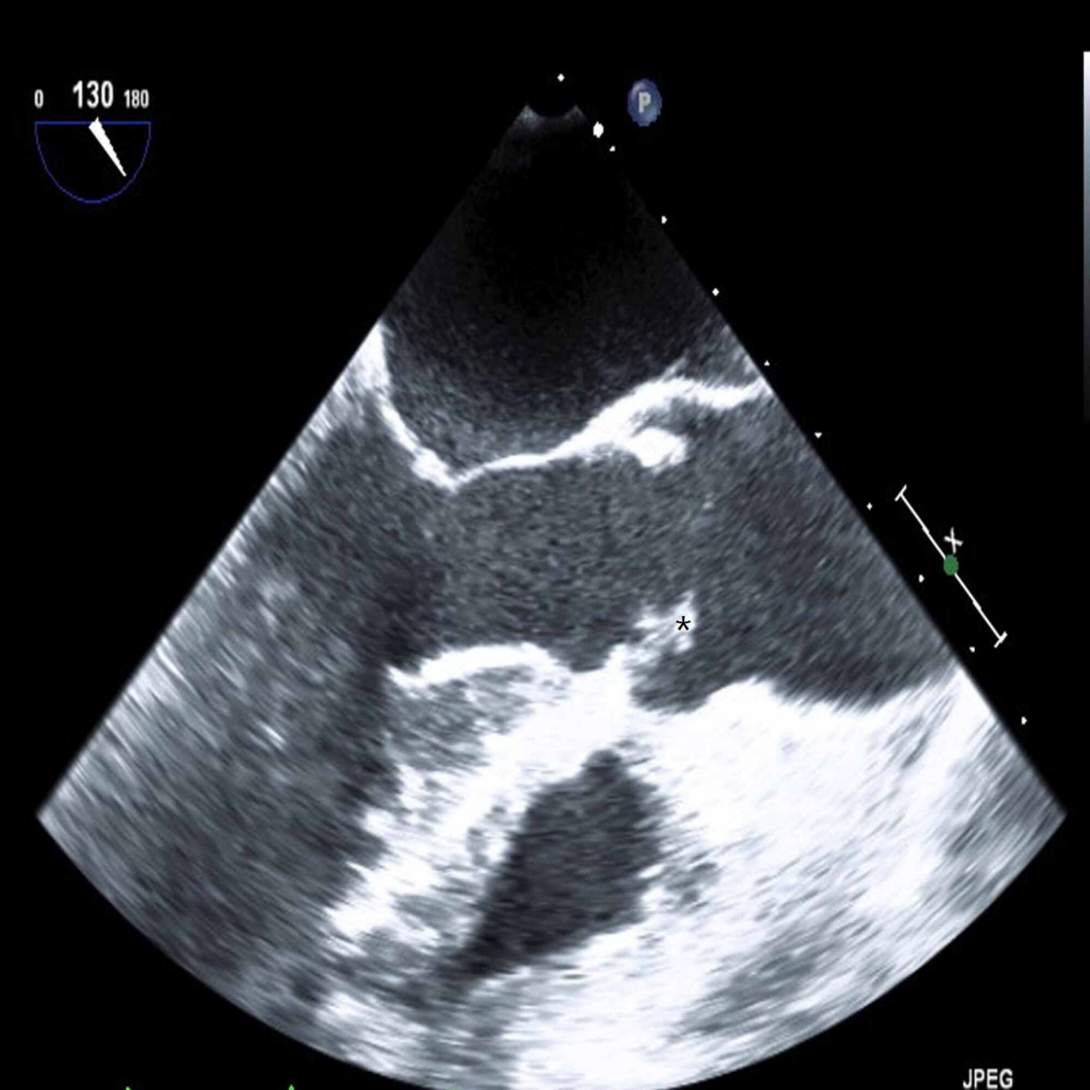
Transesophageal echocardiography with aortic long-axis view showing echo-density in aortic valve which suggestive of vegetation (black asterisk)

In the emergency department, the patient received immediate dual antiplatelet therapy, and cutaneous pacer pads were applied. Due to the focal exam findings, a computerized tomography angiogram (CTA) was performed rapidly with quick review ruling out dissection. CTA however revealed a small transverse, linear defect in the proximal aorta (Figure 2). This defect was noted to propagate towards the root of the aortic valve on its anterior leaflet suggesting possible vegetation on the aortic valve. Given the ST changes and ongoing chest pain, the patient was taken immediately for cardiac catheterization which revealed a distal thrombus in the posterior left ventricular branch of the right coronary artery which was not amenable for intervention (Figures 3, 4). During cardiac catheterization, right lower extremity pain and coolness suddenly worsened. Angiogram of the right lower extremity showed 100% occlusion of the right anterior and posterior tibial arteries (Figure 5). The patient then went for emergent thrombo-embolectomy of the right tibio-peroneal trunk, with a marked resolution of symptoms. Transesophageal echocardiography was performed later confirming the vegetation in the aortic valve measuring about 6.5x4.0 mm (Figure 6). Three sets of blood cultures were negative. Significant remarkable lab findings included C-reactive protein (CRP) of 10.5 ng/dl, erythrocyte sedimentation rate (ESR) of 60 mm/hr, and positive rheumatoid arthritis screen. Extensive rheumatological and hypercoagulable workup including anti-nuclear antibodies, complement levels, anti-thrombin III activity, protein C & S levels, homocysteine levels, and other procoagulant markers were within the normal range. Specific testing for systemic scleroderma, Sjogren’s, and polymyositis were also unremarkable. Confirmatory testing with anti-cyclic citrullinated protein showed levels of >250 units establishing the diagnosis of RA. The patient was treated with systemic oral anticoagulation and was initiated on steroids.

## Discussion

NBTE is a disease characterized by the presence of vegetations on cardiac valves, which consist of fibrin and platelet aggregates and are devoid of inflammation or bacteria [1]. As compared to the former descriptive use of marantic (“wasting away”) endocarditis, the title of NBTE is meant to encompass all forms of non-infective endocarditis including marantic endocarditis, Libman-Sacks endocarditis, and verrucous endocarditis. The majority of cases (approximately 80%) are found to be related to advanced malignancy, HIV, and SLE [2]. Other least common etiologies include other inflammatory/autoimmune disorders, including RA.

The pathogenesis in NBTE and its development is unknown, but predisposing factors include endothelial injury, hypercoagulable state, and autoimmune or inflammatory elements (via suspected cytokine-mediated damage). Particularly, the major pro-inflammatory cytokines (interleukin-6, interleukin-8, and tumor necrosis factor-α) are thought to increase the risk of thrombosis in RA patients by activation of coagulation pathways or alteration in thrombotic tendency. Moreover, patients with RA itself have been shown to have an increased risk for cardiovascular complications including, an increased risk of systemic venous thromboembolism, pericardial effusion, vasculitis, and atherosclerosis [3-5].

The relation of RA towards either NBTE or cardiovascular disease has been well documented in mainly case reports and case series but has not been investigated in large studies. Likewise, the likelihood and percentage of cases where the vegetation will embolize are also not well known. However, it has been shown that in comparison to infective endocarditis (IE), vegetations in NBTE are easily detached and can cause extensive infarction [6]. Moreover, large vegetations are of course several times more prone to embolize. Furthermore, NBTE and thromboembolic phenomena usually involve the cerebrovascular system, where the incidence of stroke in patients with NBTE is 33%, vs 19% in patients with IE. Emboli from NBTE have additionally been associated with sites that are typical to most embolic phenomena such as the brain, spleen, kidney, and peripheral arterial vasculature.

The management of NBTE includes anticoagulation and further treatment of the underlying condition. Particularly, anticoagulation should be continued indefinitely as recurrent embolization can lead to morbidity and poor prognosis, or even death as according to small retrospective studies. Likewise, recurrent thromboembolism has occurred in patients following the discontinuation of anticoagulation [7]. Of note, novel oral anticoagulants have not been studied, but older studies suggest that warfarin is less effective than heparin in reducing the rate of recurrent embolization [8]. However, warfarin may have a role in the management of patients with NBTE with extended survival in the setting of autoimmune inflammatory disorders. Regarding surgical management, the indications for surgery are the same for infective endocarditis but reports suggest that prevention of recurrent embolization is the most common reason for surgery. In general, all patients with NBTE require a thorough workup to determine the etiology as early diagnosis and treatment (including anticoagulation) can help avoid surgical removal of respective vegetations by decreasing the risk of recurrent embolism [9,10].

Given the low incidence of coronary emboli from NBTE, there is not much information from this occurrence. To the best of our knowledge, there are no reported cases of rheumatoid arthritis presenting with the acute coronary syndrome (ACS) as STEMI as well as acute limb ischemia secondary to embolic phenomena from NBTE. In our extensive review of the literature, we screened over 800+ articles through several search engines to specifically review the incidence of cases of myocardial infarction that was found to be secondary from NBTE. Of the articles included in our review, there was a total of 24 cases of NBTE causing coronary emboli and ACS [11-18]. Of these cases, 12 were STEMI, five were NSTEMI, and seven were MI that was unspecified. Of the myocardial infarctions caused by NBTE, the majority were either anterior or inferior wall territory. There are no discernable ties to mortality from anterior vs inferior STEMI. The most common cause of NBTE was noted to be secondary to malignancy followed by SLE. Both the aortic and mitral valves were the most affected in most of the cases. All patients with severe regurgitation who did not have valves replaced immediately had high mortality as compared to those who had their valve replaced. The severity of valvular regurgitation correlated more with mortality rather than the size of the vegetation. Ten out of 11 patients (90.9%) specifically with vegetation of 8 mm or larger had increased rates of embolic phenomena [19].

In the review of our patient, the workup and newly diagnosed rheumatoid arthritis coincided with her history of unspecified arthritis, as well as her, presenting and subsequent issues. Of note, blood cultures were negative on several occasions and the patient never had symptoms or clinical findings specific to an infective source. As above, RA is indeed associated with NBTE, as well as cardiovascular and thromboembolic disease. However, the prevalence of RA and STEMI secondary to NBTE, all occurring simultaneously, as occurred with our patient is rare.

## Conclusions

In conclusion, RA has been associated with increased atherosclerosis, coronary artery plaque formation, and endothelial damage. It’s extremely rare to see the presence of NBTE in RA. One must be extra vigilant especially if the size of vegetation is large as it has a tendency to embolize causing devastating complications including acute coronary syndrome and acute limb ischemia.
